# Viral afterlife: SARS-CoV-2 as a reservoir of immunomimetic peptides that reassemble into proinflammatory supramolecular complexes

**DOI:** 10.1073/pnas.2300644120

**Published:** 2024-02-02

**Authors:** Yue Zhang, Vanthana Bharathi, Tatsuya Dokoshi, Jaime de Anda, Lauryn Tumey Ursery, Nikhil N. Kulkarni, Yoshiyuki Nakamura, Jonathan Chen, Elizabeth W. C. Luo, Lamei Wang, Hua Xu, Alison Coady, Raymond Zurich, Michelle W. Lee, Tsutomu Matsui, HongKyu Lee, Liana C. Chan, Athena A. Schepmoes, Mary S. Lipton, Rui Zhao, Joshua N. Adkins, Geremy C. Clair, Lance R. Thurlow, Jonathan C. Schisler, Matthew C. Wolfgang, Robert S. Hagan, Michael R. Yeaman, Thomas M. Weiss, Xinhua Chen, Melody M. H. Li, Victor Nizet, Silvio Antoniak, Nigel Mackman, Richard L. Gallo, Gerard C. L. Wong

**Affiliations:** ^a^Department of Bioengineering, University of California, Los Angeles, CA 90095; ^b^Department of Chemistry and Biochemistry, University of California, Los Angeles, CA 9009; ^c^California NanoSystems Institute, University of California, Los Angeles, CA 90095; ^d^Department of Microbiology, Immunology & Molecular Genetics, University of California, Los Angeles, CA 90095; ^e^Biomedical Engineering, School of Engineering, Westlake University, Hangzhou, Zhejiang 310012, China; ^f^University of North Carolina Blood Research Center, Department of Medicine, University of North Carolina at Chapel Hill, Chapel Hill, NC 27599; ^g^Department of Dermatology, University of California San Diego, La Jolla, CA 92093; ^h^Division of Gastroenterology, Beth Israel Deaconess Medical Center, Harvard Medical School, Boston, MA 02215; ^i^Department of Pediatrics, School of Medicine, University of California San Diego, La Jolla, CA 92093; ^j^Stanford Synchrotron Radiation Lightsource, SLAC National Accelerator Laboratory, Stanford University, Menlo Park, CA 94025; ^k^Division of Molecular Medicine, Harbor-University of California Los Angeles Medical Center, Los Angeles County, Torrance, CA 90502; ^l^Division of Infectious Diseases, Harbor-University of California Los Angeles Medical Center, Los Angeles County, Torrance, CA 90502; ^m^Department of Medicine, David Geffen School of Medicine, University of California, Los Angeles, CA 90095; ^n^Institute for Infection & Immunity, Lundquist Institute for Biomedical Innovation, Harbor-University of California Los Angeles Medical Center, Torrance, CA 90502; ^o^Environmental Molecular Science Division, Pacific Northwest National Laboratory, Richland, WA 99354; ^p^Biological Science Division, Pacific Northwest National Laboratory, Richland, WA 99354; ^q^Division of Oral and Craniofacial Health Sciences, Adams School of Dentistry, University of North Carolina at Chapel Hill, Chapel Hill, NC 27599; ^r^Department of Microbiology and Immunology, University of North Carolina at Chapel Hill, Chapel Hill, NC 27599; ^s^McAllister Heart Institute, University of North Carolina at Chapel Hill, Chapel Hill, NC 27599; ^t^Department of Pharmacology, University of North Carolina at Chapel Hill, Chapel Hill, NC 27599; ^u^Computational Medicine Program, University of North Carolina at Chapel Hill, Chapel Hill, NC 27599; ^v^Marsico Lung Institute, University of North Carolina at Chapel Hill, Chapel Hill, NC 27599; ^w^Division of Pulmonary Diseases and Critical Care Medicine, Department of Medicine, University of North Carolina at Chapel Hill, Chapel Hill, NC 27599; ^x^Department of Pathology and Laboratory Medicine, University of North Carolina Blood Research Center, University of North Carolina at Chapel Hill, Chapel Hill, NC 27599

**Keywords:** self-assembly, antimicrobial peptides, inflammation, polyelectrolytes, superselectivity

## Abstract

It is unclear how severe acute respiratory syndrome coronavirus 2 (SARS-CoV-2) infection leads to the strong but ineffective inflammatory response that characterizes severe Coronavirus disease 2019 (COVID-19), with amplified immune activation in diverse cell types, including cells without angiotensin-converting enzyme 2 receptors necessary for infection. Proteolytic degradation of SARS-CoV-2 virions is a milestone in host viral clearance, but the impact of remnant viral peptide fragments from high viral loads is not known. Here, we examine the inflammatory capacity of fragmented viral components from the perspective of supramolecular self-organization in the infected host environment. Interestingly, a machine learning analysis to SARS-CoV-2 proteome reveals sequence motifs that mimic host antimicrobial peptides (xenoAMPs), especially highly cationic human cathelicidin LL-37 capable of augmenting inflammation. Such xenoAMPs are strongly enriched in SARS-CoV-2 relative to low-pathogenicity coronaviruses. Moreover, xenoAMPs from SARS-CoV-2 but not low-pathogenicity homologs assemble double-stranded RNA (dsRNA) into nanocrystalline complexes with lattice constants commensurate with the steric size of Toll-like receptor (TLR)-3 and therefore capable of multivalent binding. Such complexes amplify cytokine secretion in diverse uninfected cell types in culture (epithelial cells, endothelial cells, keratinocytes, monocytes, and macrophages), similar to cathelicidin’s role in rheumatoid arthritis and lupus. The induced transcriptome matches well with the global gene expression pattern in COVID-19, despite using <0.3% of the viral proteome. Delivery of these complexes to uninfected mice boosts plasma interleukin-6 and CXCL1 levels as observed in COVID-19 patients.

## Significance

At present, there are no criteria to evaluate whether a coronavirus can cause pandemics with severe inflammation or just common colds. We provide a possible answer by considering the virus not only as an infectious agent but as a reservoir of replicated peptide motifs that are not themselves pathogen associated molecular patterns (PAMPs) that specifically bind to pattern recognition receptors but are nevertheless capable of drastic immune amplification via self-assembly with PAMPs. We show evidence that viral peptide fragments from SARS-CoV-2 but not harmless coronavirus homologs can “reassemble” with dsRNA into a form of proinflammatory nanocrystalline condensed matter, resulting in cooperative, multivalent immune recognition and grossly amplified inflammatory responses.

As a result of intensive research during the COVID-19 pandemic, there now exists a working understanding of SARS-CoV-2 infection (1, 2). However, our knowledge of what makes a coronavirus a pandemic coronavirus mechanistically capable of causing a profoundly dangerous inflammatory response is still incomplete. An interesting clue was provided by a previous genomic analysis of coronaviruses, which discovered that coronavirus proteomes with high fatality rates tend to have more cationic amino acids (3), but it is not clear how altered electrostatic interactions may precipitate outcomes that characterize COVID-19. Recent work has suggested that cationic, amphiphilic peptides from the innate immune system can undergo amyloid-like assembly with anionic nucleic acids into highly proinflammatory complexes (4). Here, we take an unusual approach and consider the proteome of a coronavirus as a reservoir of peptide fragments that can be liberated upon proteolytic destruction of the virions and assess the possibility that 1) such fragments can imitate host innate immune peptides and assemble with anionic dsRNA, a ligand common in viral infections and recognized by the innate immune system (5), and 2) whether the resultant assembled supramolecular complex can conceivably be related to the diverse pathophysiology of COVID-19.

The pathophysiology of COVID-19 in the unimmunized host is indeed diverse and not well understood: Most confirmed SARS-CoV-2 cases are mild (81%), but up to 5% can develop respiratory failure, septic shock, and/or multisystem organ failure (6). Severe pulmonary inflammation is accompanied by elevated proinflammatory cytokines in serum [especially interleukin-6 (IL-6), IL-8, and tumor necrosis factor-alpha (TNF-α)] (7) and bronchioalveolar lavage (especially CXCL1, CXCL2, and CXCL6) (8), resulting in neutrophil infiltration and activation in lungs (9), consistent with development of acute respiratory distress syndrome (ARDS). Skin inflammation is manifested in the form of “COVID fingers/toes” (10). One puzzle has been the propagation of COVID-19 outcomes to multiple organs and tissues not directly infected by SARS-CoV-2 (11). For example, severe coagulation pathologies appear to be associated with endothelial dysfunction rather than direct viral infection (11). Another series of questions is centered on the occurrence of clinical arthritis-like syndromes and lupus-like syndromes in some COVID-19 patients, characterized by high autoantibody titers and immune cell activation patterns commonly seen in rheumatoid arthritis and lupus (12, 13). A fundamental understanding of COVID-19 pathophysiology needs to encompass both these serious pathologies as well as the heterogeneity of clinical severity. Although proving the specific molecular mechanisms underlying these different pathologies will require multiple large scale human studies and therefore beyond the scope of the present work, we demonstrate here that the proteome of SARS-CoV-2 exhibits a high capacity for producing peptide fragments that can lead to the seemingly disparate observations above, whereas homolog peptides from relatively harmless “common cold” viruses are much less likely to do so.

In this work, we consider the possibility that proteolytic destruction of the virus by the immune system is not necessarily the endpoint for host viral clearance in COVID-19 (in the same spirit that enzymatic breakdown of food is not the end point of food’s impact on our metabolisms). We use a machine learning classifier to help identify immunomimetic peptide sequences in the SARS-CoV-2 proteome that recapitulate functions of cationic antimicrobial peptides (AMPs) (14), a key class of effector molecules that can drive innate immune responses, the dysregulation of which can lead to autoimmune conditions such as lupus and rheumatoid arthritis. AMPs were first discovered for their antimicrobial and membrane-permeating activity, but some highly cationic AMPs such as cathelicidin LL-37 have been shown to be potently proinflammatory in lupus and rheumatoid arthritis, as well as in autoimmune skin diseases such as psoriasis and rosacea. For human LL-37 (and for AMPs with high cationic charge densities comparable to the Manning limit), this capacity for immune amplification can be traced to their ability to bind and organize anionic nucleic acids into ordered complexes via the entropy gain of counterion release. The formation of these complexes also protects the constituents from enzymatic degradation, thereby enhancing their persistence in the host and augmenting their autoimmune effects (4, 15–20). Here, we show that immunomimetic cationic SARS-CoV-2 “xenoAMPs” can chaperone and organize anionic poly(I:C), a synthetic analog of dsRNA, into liquid crystalline complexes with lattice constants that are commensurate with the steric size of dsRNA receptor TLR3 (Toll-like receptor-3), which enhances cooperative electrostatic multivalent binding and amplification of immune activation (20, 21) via a variation of superselectivity, which was originally conceived for multivalent interactions in nanoparticles (22). Together with cognate effects from “immune vetting” of supramolecular complexes, a grossly distorted immune response can in principle be provoked (23, 24), especially given the large numbers of virions available in hosts with serious infections (25). Consistent with this hypothesis, we show that xenoAMP-poly(I:C) complexes trigger strong cytokine secretion in a broad range of healthy, uninfected cells, including epithelial cells, endothelial cells, monocytes, and macrophages in culture. The transcriptome of primary endothelial cells activated by xenoAMPs matches well with the global gene expression profile in COVID-19 infections, even though the peptide fragments used comprise less than 0.3% of the viral proteome. Delivery of these complexes to mice boosts plasma IL-6 and CXCL1 levels as observed in humans with COVID-19 (7, 26). These results suggest an unanticipated mechanism for severe COVID-19 derived pathologies such as cytokine storms, skin lesions, coagulation disorders, and significantly impact tissues that are not the direct target of infection.

## Results

The effect of viral AMP-like fragments on host cells will depend on the types and available numbers of such molecules. Therefore, we map out and parse all AMP-like sequences (xenoAMPs) available in the SARS-CoV-2 proteome. To determine the full scope and nature of xenoAMPs, a number of questions need to be considered. This determination is compounded by unusually large size of the RNA genome of SARS-CoV-2 (~30 kilobases, kb), which encodes ~30 mature proteins (27) that are heavily processed by host and/or viral proteases to produce essential functional moieties. Are there strong AMP-like motifs in the SARS-CoV-2 proteome, and how many are there? Given that the numbers of virions are large in an infected host (25), it is especially important to see whether xenoAMPs are found in repeating structures such as the spike protein. How sensitive is the AMP-like function encoded within these peptide motifs to cleavage at slightly different positions by different proteases? Finally, how do these AMP-like motifs change if we analyze low pathogenicity, “common cold” coronaviruses?

To answer these questions, we use a previously trained support vector machine (SVM) classifier to recognize AMP-like sequences (28, 29) in SARS-CoV-2 proteins. This classifier has been validated in a broad range of systems (30–32). First, to identify potential xenoAMPs and assess whether they are still AMP-like if cleaved at different nearby amino acid positions, SARS-CoV-2 protein sequences are scanned via a moving window of 24 to 34 amino acids, a typical size of many AMPs. In contrast to traditional bioinformatic tools, this approach can reveal AMP-like sequences with low sequence similarity to known AMPs, and thus ideally suited for identification of unanticipated proinflammatory xenoAMP candidates in SARS-CoV-2. The classifier outputs a sigma score (σ) that characterizes the AMP-ness of a given sequence: Strongly positive and strongly negative σ scores indicate high probabilities *P*(+1) of the sequence being an AMP or not being an AMP, respectively. (A σ score higher than 0 corresponds to a probability of *P*(+1) > 0.5). From this population of high-scoring AMP-like sequences, we select specific sequences with sufficiently high cationic charge to mimic the capacity of human cathelicidin LL-37 to organize anionic dsRNA into ordered structures for immune modulation^*^, since dsRNA is a pathogen-associated molecular pattern expected to be released from damaged cells in SARS-CoV-2 infections.

**Fig. 1.**
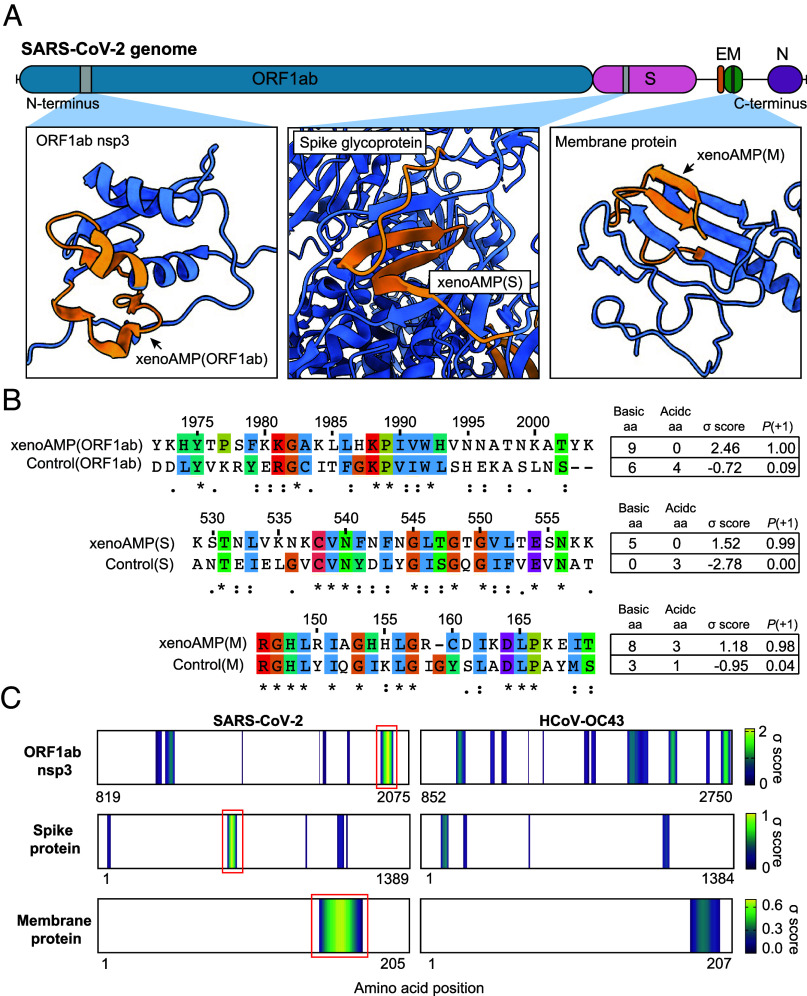
Existence of exogenous mimics of pro-inflammatory host antimicrobial peptides (xenoAMPs) in SARS-CoV-2 proteins. (*A*) SARS-CoV-2 proteins are scanned with a machine learning AMP classifier. Each queried sequence is given a σ score that measures its AMP-ness. Three representative high-scoring sequences are studied: xenoAMP(ORF1ab), xenoAMP(S) and xenoAMP(M). The grey bars mark the location where the corresponding sequences are selected. (*B*) SARS-CoV-2 sequences are aligned and compared to their homologs in a common cold human coronavirus HCoV-OC43: Control (ORF1ab), Control(S) and Control(M). Asterisks, colons, and periods indicate positions that have fully conserved residues, those that have strongly similar properties, and those that have weakly similar properties, respectively. Color is assigned to each residue using the ClustalX scheme. (*C*) σ score heatmaps compare the distribution of high scoring sequences in three proteins from SARS-CoV-2 and HCoV-OC43. The first amino acid in each sequence is colored according to its average σ score; regions with negative average σ scores (non-AMPs) are colored white. “Hot spot” clusters of high-scoring sequences for SARS-CoV-2 (bright yellow regions bracketed in red boxes) have systematically higher scores and span wider regions of sequence space compared to HCoV-OC43. This trend suggests that hot spots in SARS-CoV-2 can generate higher scoring sequences for a greater diversity of enzymatic cleavage sites than those in HCoV-OC43.

Although the spatiotemporal distribution of viral fragments in individual hosts will be varied, the fundamental availability of strong xenoAMP sequences is rooted in the viral proteome itself. We focus on prototypical candidates with high scores and high cationic charge from three representative proteins (one nonstructural, two structural; selection criteria σ score > 1.1, *P* (+1) > 0.98): xenoAMP(ORF1ab) from the ORF1ab polyprotein (1972 to 2004, σ score = 2.46), xenoAMP(S) from the spike (S) protein (529 to 558, σ score = 1.52), xenoAMP(M) from the membrane (M) protein (146 to 169, σ score = 1.19) (Fig. 1*A*). Consistent with AMP-like behavior, broad-spectrum xenoAMP antimicrobial activity has been observed using radial diffusion assays (RDA) against both Gram-positive and Gram-negative bacterial strains, including a Methicillin-Resistant *S. aureus* (MRSA) strain (LAC-USA300) and *Pseudomonas aeruginosa* PA01 (*SI Appendix*, Table S1). In silico analysis of cleavage sites on SARS-CoV-2 proteins indicates that xenoAMPs can be produced during proteasomal degradation. (*SI Appendix*, Fig. S1) Our result show that neutrophil elastase (NE) and matrix metalloproteinase-9 (MMP-9) are both capable of generating xenoAMPs. We note that high expression levels of these two specific proteases have been correlated to acute lung injury and hyperinflammation (33).

We directly compare these xenoAMPs from SARS-CoV-2 with the homologous sequences from human coronavirus-OC43 (HCoV-OC43), a mild common cold coronavirus (Fig. 1*B*). More detailed comparisons between aligned sequences in highly pathogenic human coronaviruses (SARS-CoV-2 and SARS-CoV) and mild, nonpandemic human coronaviruses (HCoV-HKU1, HCoV-OC43, HCoV-229E, and HCoV-NL63) show that the sequences are partially conserved, but with the more pathogenic coronaviruses, especially in SARS-CoV-2 and SARS-CoV, having higher σ scores that better mimic host cationic AMPs (*SI Appendix*, Fig. S2). Comparison of SARS-CoV-2 vs. HCoV-OC43 σ score heat maps for the ORF1ab polyprotein, the S protein, and the M protein (Fig. 1*C*) shows that high-scoring sequences are generally clustered into “hotspots” at different locations, with the SARS-CoV-2 hotspots having systematically higher scores and spanning wider regions of sequence space compared to HCoV-OC43 (hotspots are more yellow than blue and have larger footprints). This analysis indicates that the AMP-ness of specifically SARS-CoV-2 xenoAMPs can be quite persistent with varying degrees of cleavage: The cationic and hydrophobic amino acids in sequences of the SARS-CoV-2 proteins are organized so that σ scores remain surprisingly high even with imperfect cleavage events that lengthened or shortened the sequences relative to the high-scoring exemplar xenoAMPs. (*SI Appendix*, Table S2) Together, these lines of evidence suggested that hot spots in SARS-CoV-2 can generate more xenoAMPs with higher scores and that xenoAMPs from SARS-CoV-2 are less vulnerable to having their scores lowered by length heterogeneity from degradative processes compared to those from HCoV-OC43. What’s more, that the availability of xenoAMPs from SARS-CoV-2 is amplified by the higher transmissibility and faster replication kinetics of SARS-CoV-2 compared to HCoV-OC43 can further contribute to larger numbers of xenoAMPs in human host with COVID-19 (34, 35).

Compared to ‘common cold’ coronaviruses, we note that SARS-CoV-2 has more sequences with both high cationic charge and high machine learning scores. These sequences are optimal for mimicking host proinflammatory AMPs like LL-37, which bears a high linear charge density of ~+1.1e/nm. The LL-37 charge density approaches the Manning limit of 1 charge per Bjerrum length (0.7 nm), the idealized criterion for linear macroions to sequester condensed counterions and therefore capable of strong entropically driven complexation with dsRNA. Since cationic charge is a fundamental property of AMPs, our results are consistent with previous whole genome analysis finding positive charge accumulation in coronaviruses with high fatality rates (3). To summarize, SARS-CoV-2 has xenoAMP sequence candidates that have systematically higher machine learning σ scores, and have high probabilities of functioning like AMPs. Moreover, these xenoAMP candidates have higher cationic charge, which is conducive to proinflammatory dsRNA organization. Finally, high-scoring xenoAMP candidates can in principle be generated by more diverse enzymatic cleavage sites in SARS-CoV-2 compared to those from the nonpandemic coronavirus HCoV-OC43. This last point is important given that the host harbors a broad range of proteases with different cleavage sites at various tissues.

Cationic AMPs have a strong tendency to strongly bind anionic lipids and anionic nucleic acids because such binding is related to their charge and to their known antimicrobial mechanisms (14, 36, 37). Therefore, in general, free AMPs are not expected to be detected at high concentrations in vivo. Nevertheless, we performed mass spectrometry on tracheal aspirate samples from 29 severe COVID-19 patients to assess what can be detected using a bottom–up proteomics data collection protocol that prioritizes unbiased detection. Neutrophilia from inflammation in severe COVID-19 is expected to result in the release of host AMP LL-37. Consistent with this expectation, we find fragments of LL-37 in 20 out of 29 patient samples from mass spectrometry measurements. In comparison, we find SARS-CoV-2 peptide fragments in 28 out of 29 patient samples, with some fragments having high σ scores to be classified as xenoAMPs themselves. Details of the measurement and analysis are presented in *SI Appendix*, Fig. S3.

**Fig. 2.**
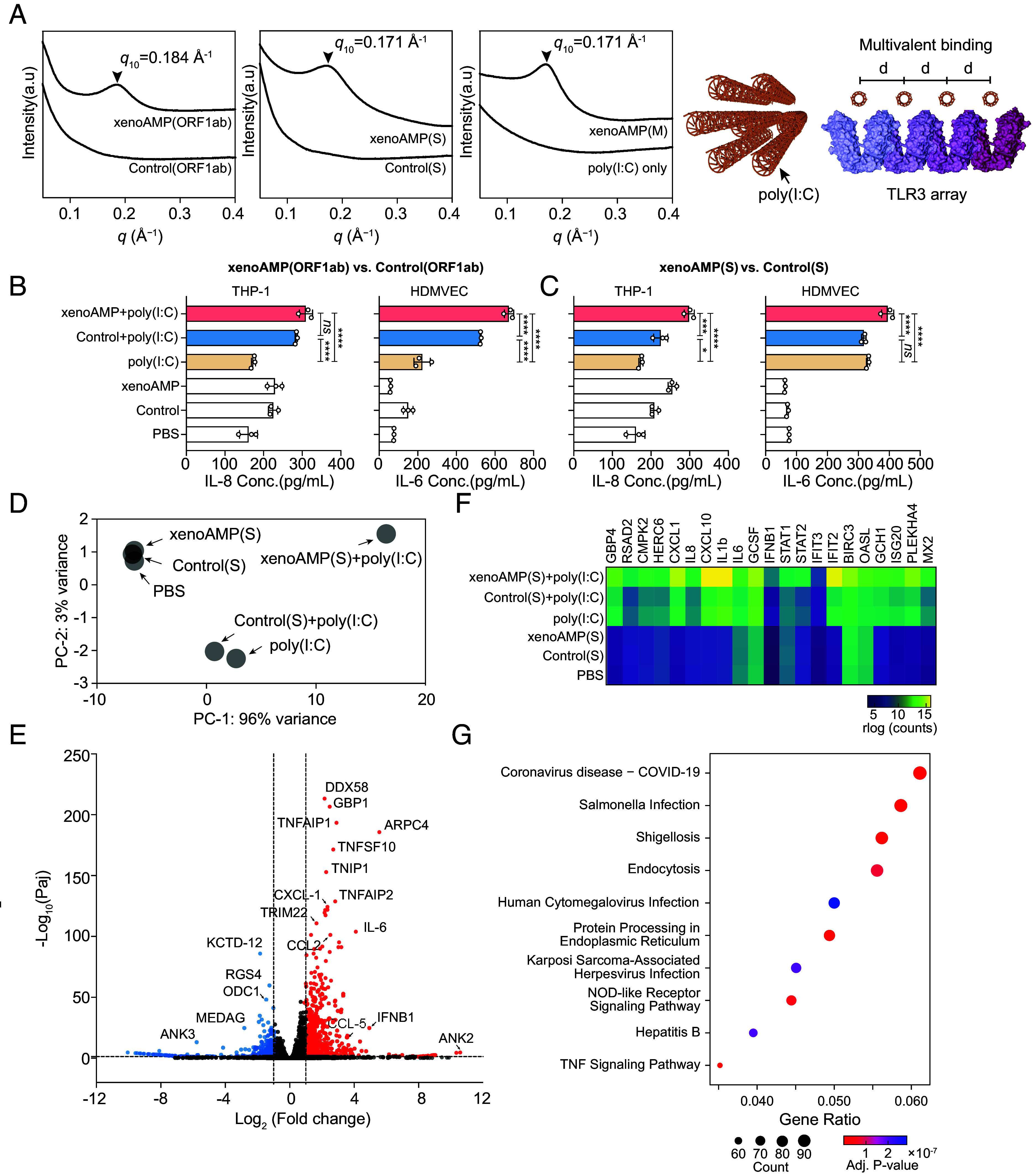
XenoAMPs from SARS-CoV-2 but not homologs from HCoV-OC43 organize poly(I:C) into ordered complexes that amplify immune responses. (*A*) SAXS data show that SARS-CoV-2 derived complexes exhibit a liquid crystalline structure that can amplify activation of TLR3, whereas HCoV-OC43 complexes do not. (*B* and *C*) Cytokine release from THP-1 monocytes and human dermal microvascular endothelial cells (HDMVEC) that are stimulated with SARS-CoV-2 xenoAMP-poly(I:C) complexes or the HCoV-OC43 Control-poly(I:C) complex (n = 3). Data is presented as mean ± SE. Statistical analysis is performed using one-way ANOVA (*P < 0.05, **P < 0.01, ***P < 0.001, ****P < 0.0001). (*D*) Principal component analysis of the transcriptome profile in HDMVEC treated by xenoAMP(S)-poly(I:C) complexes, Control(S)-poly(I:C), poly(I:C) only and associated controls. (*E*) Differential gene expression in HDMVEC treated by xenoAMP(S)-poly(I:C) compared to the poly(I:C) treatment group. Significance (−Log_10_(Paj), *y* axis) is plotted against magnitude of gene expression (Log_2_(Fold change), *x* axis). Significantly upregulated and downregulated genes (Paj < 0.05, Log_2_(Fold change) > 1; Log_2_(Fold change) <−1) are highlighted in red and blue respectively. (*F*)Transcriptional change of immune-related genes in HDMVEC in different conditions. (*G*) By comparing all significantly upregulated genes (*P* < 0.05) to curated expression patterns from the KEGG database, the best match to the gene expression pattern induced by complexes with the xenoAMP(S) fragment is COVID-19 (*P* < 1 × 10^−7^).

Consistent with the machine learning-based analysis, all three high-scoring, high cationic charge xenoAMPs were experimentally found to chaperone and organize dsRNA into ordered complexes in a manner cognate to that exhibited by AMPs like human cathelicidin LL-37 (21), even though these xenoAMPs exhibit less ordered structures than typical AMPs. LL-37 assembles nucleic acids into a nanocrystalline columnar lattice with an internucleic acid spacing in the ~3.3 to 4.0 nm range, which allows multivalent presentation to close-packed TLR3 (for dsRNA) and TLR9 (for dsDNA), resulting in drastically amplified immune activation (20, 38). To mimic viral dsRNA produced during viral replication, poly(I:C) is used as a synthetic analog in the following experiments. The structures of all three isoelectric xenoAMP-poly(I:C) complexes show the same basic diffraction signature in synchrotron Small Angle X-ray Scattering (SAXS): For xenoAMP(ORF1ab)-poly(I:C), xenoAMP(S)-poly(I:C), and xenoAMP(M)-poly(I:C) complexes, we observe unambiguous diffraction peaks that correspond to liquid crystalline ordering with inter-dsRNA correlations of 3.41 nm (*q* = 0.184 Å^−1^), 3.67 nm (*q* = 0.171 Å^−1^), and 3.67 nm (*q* = 0.171 Å^−1^) respectively (Fig. 2*A*). The structures of these xenoAMP-poly(I:C) complexes are cognate to those of host AMP-dsRNA complexes, with all inter-dsRNA spacings in the range of values (*q*_10_ = 0.17 to 0.19 Å^−1^) that correspond to strong immune activation. We compare the above results to those from the homolog sequence of xenoAMP(ORF1ab) (Control(ORF1ab)) and xenoAMP(S) (Control(S)) in human coronavirus HCoV-OC43, a strain that causes common colds. SAXS correlation peaks are strongly suppressed in Control(ORF1ab)-poly(I:C) and Control(S)-poly(I:C) complexes, implying much weaker capacity for inducing immune activation.

Given the variation of infection and immune responses in individuals, it is informative to evaluate how robust the above self-assembled proinflammatory structures are when conditions are not optimal. We examined how much the self-assembled structures change when the peptide length deviates from high-scoring exemplar xenoAMP sequences. We also examine how the self-assembled structures are influenced by peptide population heterogeneity. Consistent with the observed robustness of AMP-ness (measured by the σ score) with peptide length variation, we found that the nanocrystalline structure of SARS-CoV-2 poly(I:C) complexes is preserved when the participating xenoAMPs are shorter than the main exemplars studied here (*SI Appendix*, Fig. S4*A*). In an infection scenario, we expect a diverse population of xenoAMPs from the virus to coexist with one another and with host AMPs such as LL-37. As an extreme case of heterogeneity, we examine how SARS-CoV-2 xenoAMPs interact with poly(I:C) in the presence of LL-37. The data show that surprisingly, SARS-CoV-2 xenoAMPs can cocrystallize with LL-37 into the same columnar lattice, which suggest that xenoAMPs and host AMPs can in principle work synergistically in activating inflammatory responses (*SI Appendix*, Fig. S4 *B* and *C*).

We compared the capacity of SARS-CoV-2 xenoAMPs and the homolog peptide from HCoV-OC43 for immune activation in vitro. Monocytes serve as an essential antiviral defense mechanism, but their dysregulation in COVID-19 (39, 40) results in hyperactive cytokine release, cytotoxic cell infiltration, and tissue damage. We found that xenoAMP-poly(I:C)-treated human monocytic THP-1 cells release ~1.7-fold more IL-8 compared to the poly(I:C)-only control (Fig. 2 *B* and *C*). In contrast, the complexes formed with the homolog sequences of the low pathogenic common cold HCoV-OC43 viral strain induce much lower IL-8 release. Recent studies show that inflammatory monocytes in the lung are highly enriched with SARS-CoV-2 RNA, which can in principle further amplify the proinflammatory cytokine release in vivo (41).

Skin lesions in the form of COVID fingers/toes and other rashes are common immunopathologies in COVID-19 and the associated multisystem inflammatory syndrome in children (MIS-C) (42). We stimulate primary human dermal microvascular endothelial cells (HDMVEC) with xenoAMP-poly(I:C) complexes and find strong IL-6 production, elevated by 2.2× to 3.1× compared to poly(I:C) control, but not with complexes formed with homolog peptide from HCoV-OC43 (Fig. 2 *B* and *C*). Moreover, up-regulated transcription levels of IFNB1, IFNA2, STAT1, and STAT2 suggest activation of the interferon signaling pathway (*SI Appendix*, Fig. S5). In additional experiments with primary Normal Human Epidermal Keratinocytes (NHEK), we find that xenoAMP-poly(I:C) complexes induce 7.9× higher C-X-C motif chemokine 10 (CXCL10) release and 13.9× higher IL-6 release compared with poly(I:C) treatment alone. Similar levels of immune activation were observed for LL-37-poly(I:C)-treated NHEK cells, suggesting that SARS-CoV-2 xenoAMPs can act like host natural AMPs in immune vetting (*SI Appendix*, Fig. S6) The augmentation of cytokines release in endothelial cells and keratinocytes can promote microvasculature via release of vessel formation factors, which in turn correlate with skin inflammation.

Compared to poly(I:C)-only control, xenoAMP(S)-poly(I:C) complex-treated HDMVEC exhibited a distinct transcriptome profile with significantly up-regulated transcription of proinflammatory cytokine and chemokine-related genes, including CXCL1, IL8, CXCL10, IL1b, IL6, GCSF (granulocyte colony-stimulating factor), IFNB1, and others (Fig. 2 *E* and *F*). These are consistent with the clinical features associated with the COVID-19 cutaneous manifestations, featured by perivascular lymphocyte infiltration in the cases with intermediate severity and leukocytoclastic vasculitis in the highly severe cases (43). Interestingly, the five cytokines (CCL2, CCL3, CCL20, CXCL1, and IL8) that are involved in the leukocyte infiltration in the blood vessel of rheumatoid arthritis patients are also found in the xenoAMP(S)-poly(I:C) activated HDMVEC. Two of three established markers for severe COVID-19 illness in our library (44), IL-8 and GCSF, are up-regulated more than 2× compared to dsRNA-only control. Next, we match all differentially expressed genes (*P* < 0.05) to signaling pathways from the Kyoto Encyclopedia of Genes and Genomes database (KEGG Enrichment) to see how gene expression compares to those curated from known pathologies. Though we use a specific cell type (HDMVEC) to assess tissue-specific effects, the best match to the observed global gene expression pattern was COVID-19 itself (*P* < 1 × 10^−7^, Fig. 2*G*). This represents a form of “viral synecdoche”, through which a SARS-CoV-2 fragment that constitutes <0.3% of the viral proteome significantly recapitulated COVID-19 gene expression patterns from infection by the intact SARS-CoV-2 virion. Genes for NOD-like receptor signaling and TNF signaling-related pathways are likewise up-regulated. By comparison, the transcriptome profile in Control(S)-poly(I:C)-treated HDMVEC resemble that of the poly(I:C) only stimulation, consistent with knowledge that infection with the common cold coronavirus HCoV-OC43 seldom causes multisystem inflammation (Fig. 2*D*).

**Fig. 3.**
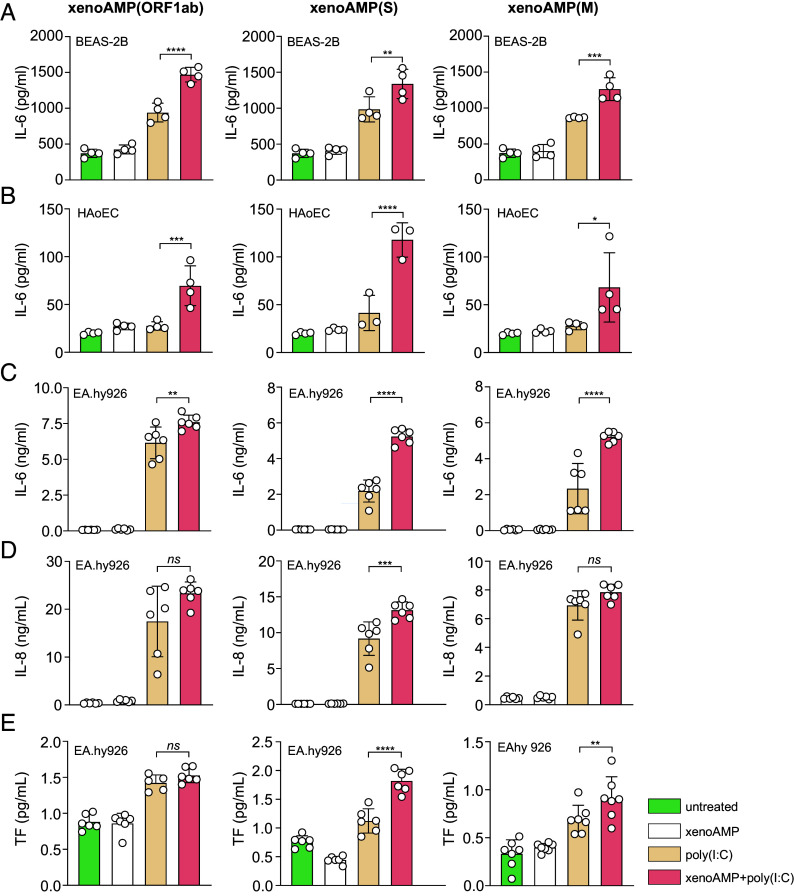
xenoAMPs-poly(I:C) complexes activate inflammation and coagulation processes. Bronchial epithelial cells (BEAS-2B, n = 4, *A*), primary Human Aortic Endothelial Cells (HAoEC, n = 4, *B*) and human umbilical vein cells (EA.hy926, n = 6, *C* and *D*) are treated with xenoAMP-poly(I:C), poly(I:C) or the associated controls. The released cytokines (IL-6 and IL-8) in the supernatant are quantified to evaluate the immune activation. The tissue factor (TF) activity is measured by a two-stage Factor Xa activity assay. (n=6, *E*) Representative results are shown from at least three independent experiments. Data is presented as mean ± SD. The statistical analysis is performed using one-way ANOVA. (*P < 0.05, **P < 0.01, ***P < 0.001, ****P < 0.0001).

Generality of xenoAMP-poly(I:C) complex-driven immune activation observed above has been investigated also on human lung bronchial epithelial cells (BEAS-2B), primary Human Aortic Endothelial Cells (HAoEC), and human vein endothelial cells (Ea.hy926). Cells treated with xenoAMPs-poly(I:C) complexes release higher levels of IL-6. Compared to poly(I:C)-only treatment (baseline activation level with dsRNA only but no xenoAMP amplification), xenoAMP-poly(I:C) treatment leads to ~1.4 to 1.6× increase for BEAS-2B cells (Fig. 3*A*), 2.4 to 2.8× increase for HAoEC cells (Fig. 3*B*), ~1.2 to 2.4× increase for Ea.hy926 cells (Fig. 3*C*). Ea.hy926 IL-8 levels are increased ~1.4× in the xenoAMP(S)-poly(I:C) treatment group but not significantly increased for the xenoAMP(ORF1ab)-poly(I:C) and xenoAMP(M)-poly(I:C) treatment groups, suggesting existence of xenoAMP species-specific effects (Fig. 3*D*). In Ea.hy926 endothelial cells, xenoAMPs-poly(I:C)-induced TLR3-mediated tissue factor (TF) production can in principle be related to observation of amplified coagulation in COVID-19 (Fig. 3*E*).

**Fig. 4.**
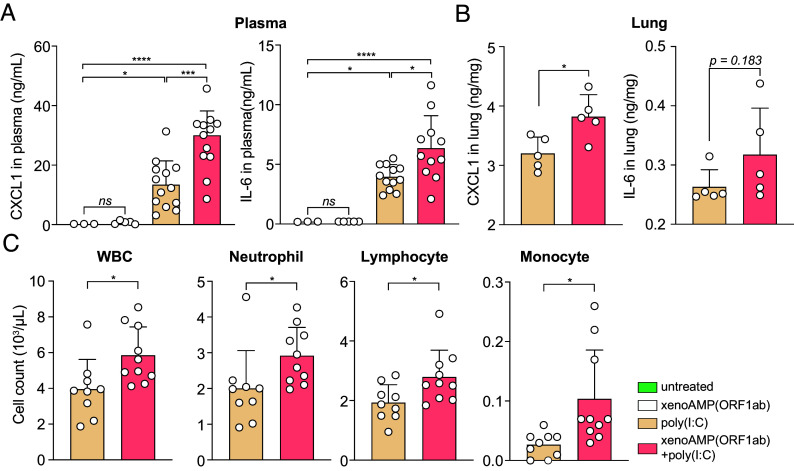
xenoAMP(ORF1ab)-dsRNA complex activate inflammation in C57BL/6 mice. C57BL/6 mice is either untreated or injected with xenoAMP(ORF1ab), poly(I:C) or xenoAMP(ORF1ab)-poly(I:C) complex. Samples are collected after 2 h. (*A*) Concentration of CXCL1 and IL-6 in the plasma. (*B*) Concentration of CXCL1 and IL-6 in the lung (n = 5). (*C*) WBC, neutrophil, lymphocyte and monocyte count in the peripheral blood (n = 10). All the data is presented as mean ± SD. The statistical test of the hematology data and lung cytokine data are performed with unpaired two-tailed *t* test, while the plasma cytokine data is analyzed with one-way ANOVA (*P < 0.05, **P < 0.01, ***P < 0.001, ****P < 0.0001).

We measure the capacity of xenoAMPs- poly(I:C) complexes for immune activation using a mouse model. (Immune activation of murine bone marrow–derived macrophages (BMDM) is shown in *SI Appendix*, Fig. S7) XenoAMP(ORF1ab)-poly(I:C) complexes and associated controls are administered intravenously to healthy C57BL/6 mice that have not been exposed to viral infection. Compared to poly(I:C)-only treatment, xenoAMP(ORF1ab)-poly(I:C) treatment strongly increase plasma IL-6 and CXCL1 levels by 1.6× and 2.2× respectively. (Fig. 4*A*) Immune activation by xenoAMPs-poly(I:C) complexes is evaluated specifically in the lung, which is strongly affected in COVID-19 patients (41). CXCL1 and IL-6 levels due to xenoAMP(ORF1ab)-poly(I:C) increase by 1.2× compared to poly(I:C)-only stimulation. (Fig. 4*B*) CXCL1 is a chemoattractant for neutrophils, the infiltration of which in the lungs can drive ARDS in COVID-19. IL-6 is a proinflammatory cytokine, and high plasma levels of IL-6 serve as a strong predictor for nonsurvival in COVID-19 patients (7). Importantly, xenoAMP(ORF1ab)-poly(I:C) complex significantly increases the cell count for white blood cells (WBCs), which may be related to the enrichment of neutrophils, lymphocytes, and monocytes (Fig. 4*C*). These results also suggest that inflammatory responses are xenoAMPs-dependent and heterogeneous, even for genetically identical mice, and that future in vivo comparisons using xenoAMPs from the many new variants and the many nonpandemic coronaviruses may be fruitful.

## Discussion

We have shown that an unanticipated mechanism for propagating inflammation through uninfected cells exists for SARS-CoV-2 but not for common cold coronaviruses. This mechanism involves viral fragments able to mimic AMPs like LL-37 cathelicidin in host innate immunity. Given that LL-37 is involved in pathogenesis of lupus and rheumatoid arthritis, the notion that SARS-CoV-2 peptide fragments can imitate LL-37 may be conceptually salient for understanding why the immune systems of COVID-19 patients resemble those of people with autoimmune disorders like lupus and rheumatoid arthritis (45).

The concept of COVID-19 propagation in the host via viral peptide fragments in addition to direct tissue infection creates many potential points of contact with existing observations. The formation of complexes between such peptides and nucleic acid is interesting from the perspective of hyperinflammation and autoimmune reactions. Clinical studies find that extrapulmonary multisystem pathologies precipitated by SARS-CoV-2 pneumonia may be related to the massive release of cell debris (nucleic acids and protein fragments of viral as well as host origin) into the circulatory system. The thermodynamic driving force for complex formation between xenoAMPs and nucleic acids is large, given that the magnitudes of their cationic and anionic surface charge densities are unusually close, which leads to large counterion entropy gains upon binding. Such supramolecular complexes can in principle be endocytosed by immune cells and nonimmune cells alike. The inter-nucleic acid distance in these complexes are close to those from LL-37-nucleic acid complexes implicated in lupus and rheumatoid arthritis, thus allowing for optimal multivalent presentation of nucleic acid to endosomal TLRs. Such aberrant TLR activation (which leads to increased levels of IL-6 and TNFα) can result in amplified and prolonged inflammatory responses that desynchronize with viral clearance. Similarly, the inappropriate self-nucleic acid sensing by TLRs can also disrupt immune tolerance to self-nucleic acids in a manner similar to autoimmune disorders. Studies have found that TLRs activation in COVID-19 patients is correlated with disease severity (46). TLR antagonists have recently entered clinical trials. (e.g., Merck M5049 for treating COVID-19 pneumonia).

The proinflammatory impact of SARS-CoV-2 xenoAMPs documented here highlights the potential role of proteases in COVID-19 pathogenesis. From our in silico prediction, NE and MMP-9 are both capable of generating xenoAMPs (*SI Appendix*, Fig. S1). In addition to direct damage to host tissue, up-regulated expression of NE and MMP-9 (47, 48) in critically ill COVID-19 patients can contribute to the proliferation of proinflammatory xenoAMPs. In a more general compass, it is interesting that recent work suggests that protease activity can lead to severe inflammation in other contexts (49, 50). These observations suggest that an appropriate combination of host protease inhibitors that suppress xenoAMP formation may have a clinical impact on suppressing severe SARS-CoV-2-driven inflammation. Serine proteases used in the host innate immune system for making AMPs may be good initial target candidates.

Interestingly, proteolytic degradation of SARS-CoV-2 is likely to be heterogeneous, as individual hosts display distinctive patterns of enzyme efficiencies varying routinely by fourfold to 50-fold (51), with protein expression being “noisy” even at the single cell level (52). Stochastic transcription or alternative splicing of mRNA especially impacts environmentally responsive proteins (like proteases) (53) with expression especially variable among genes of innate immunity (54). That proteolytic degradation of SARS-CoV-2 is expected to be drastically different among hosts may explain why the infection outcomes of SARS-CoV-2 are so heterogeneous, ranging from asymptomatic hosts to fatalities.

It is clear that xenoAMPs can result in a broader range of pathologies than the in vitro and in vivo results presented here, especially since mass spectrometry, machine learning, and protease cleavage analysis results all point to the existence of a heterogeneous distribution of SARS-CoV-2 xenoAMPs. Although these observations indicate that identification of xenoAMP-nucleic acid complexes with specific individual species of xenoAMP is not statistically practical, the prevailing understanding of electrostatics in aqueous media (55, 56) suggests that because of counterion entropy, peptides such as LL-37 and SARS-CoV-2 xenoAMPs will preferentially bind nucleic acids such as dsRNA since they have surface charge densities that are opposite in sign and importantly, comparable in magnitude. This effect underpins one of the key differences between pandemic and nonpandemic coronaviruses: Homolog sequences from common cold coronavirus HCoV-OC43 do not form proinflammatory complexes (Fig. 2*A*), even though some of them have multiple cationic charges to interact with anionic dsRNA. In fact, nucleic acid complexes formed with LL-37 or SARS-CoV-2 xenoAMPs are expected to be unusually stable in physiological environments due to counterion osmotic pressure (56, 57). Indeed, LL-37-nucleic acid complexes have been observed in lupus patients (17) and in COVID-19 patients (58). These complexes exhibit structures that are stable in serum conditions (38). These observations are not surprising given that such complexes are spontaneously assembled and held together by similar interactions that stabilize nucleic acids complexes used in nonviral gene therapy.

Given the observation of severe inflammation in COVID-19, one intriguing question is how the existence of host AMPs from innate immunity may add to xenoAMP activity. LL-37-poly(I:C) complexes can induce quantitatively higher immune activation compared to xenoAMP-poly(I:C) complexes. Average IL-6 levels in LL-37-poly(I:C) complex-treated NHEK cells is ~2× higher than that for xenoAMP(S)-poly(I:C) complex-treated NHEK cells. It is possible that LL-37 is superior to xenoAMPs in mediating cell entry of the complexes. What is interesting and unanticipated is the extent to which LL-37 can combine with SARS-CoV-2 xenoAMPs and cooperatively produce more ordered dsRNA complexes that are potentially more effective in inducing inflammation than either LL-37 or xenoAMPs alone (*SI Appendix*, Fig. S4 *B* and *C*). These results need to be tested in vivo but may ultimately connect to vulnerability of patients with preexisting inflammatory conditions to COVID-19.

The formation of complexes between viral peptide fragments and nucleic acids can have longer-term consequences beyond acute inflammation since both the peptide and nucleic acid components are protected against enzymatic degradation in the host. What’s more, some of the xenoAMP sequences have some propensity to fold into either α-helical or β-sheet structures. Moreover, a recent study has shown that fragments from NE processed SARS-CoV-2 spike proteins can form amyloids, which in principle can contribute to greater longevity in vivo (59). More generally, it is known that the existence of viral remnants in the host is associated with chronic disease in influenza infections (60). Viral RNA has been found to persist in various body fluids of individuals recovered from Ebola infections for more than a year (61, 62). It is possible that complex formation by SARS-CoV-2 xenoAMPs and nucleic acids can, at least in part, account for longer-term effects of COVID-19.

Results presented here indicate that there exist intrinsically proinflammatory sequences found in the SARS-CoV-2 proteome that are not found in common cold coronavirus homologs, sequences that strongly activate immune responses in a broad range of cell and tissue types connected to disease states in multiple systems. The present study has a number of limitations. Given that the strong proinflammatory activity of xenoAMP-dsRNA complexes has been confirmed in an uninfected mouse model here, it will be informative to assess in a full SARS-CoV-2 infection animal model whether the small population with severe inflammatory syndromes [5% of infected human hosts developed into critical state (6)] has increased protease activity for generating xenoAMPs and/or harbor a detectable residual distribution of peptide-dsRNA complexes that contain diverse SARS-CoV-2 xenoAMP sequences capable to induce immune outcomes. It will also be useful to investigate the temporal persistence and heterogeneity of this mode of immune activation via a kinetic study of infection-induced inflammation outcomes. Since the transcriptome from HDMVEC’s stimulated with peptide-dsRNA complexes with even a single xenoAMP species can recapitulate gene expression from COVID-19, it will be illuminating to see how different xenoAMP species act in concert. Moreover, it will be informative to see how the gene expression induced by xenoAMP-dsRNA complexes varies for different cell types in general.

Looking beyond the effects of xenoAMP-dsRNA complexes, it will be important to examine more broadly the effects from SARS-CoV-2 xenoAMPs alone, without complexation with dsRNA. It is quite possible that AMP-like viral fragments generated ectopically can be harmful, especially to vulnerable tissues such as the endothelium. potential downstream effects range from loss of hemostatic regulation (e.g., hyperpermeability of vasculature), lysis of specific immune cell types, and intensification of cytokine storms, which are reminiscent of COVID-19 symptoms (63).

Taken together, the results presented here suggest an unanticipated mode of amplified immune activation for healthy, uninfected cells without direct infection by SARS-CoV-2, in addition to inflammation resulting from direct infection. That xenoAMP-dsRNA complexes can induce these inflammatory effects is surprising, since binding and “sequestration” of immune ligands are usually associated decreased and not drastically increased levels of immune activation. The methods outlined here based on the structure and phase behavior of dsRNA complexes with coronavirus xenoAMPs can in principle assess the inflammatory potential of the large number of new variants of interest, such as the diverse Omicron variants. The results presented here suggest that viral fragments can connect to a broad range of seemingly unrelated COVID-19-associated pathologies, such as cytokine release syndrome, coagulation disorders, skin lesions, including potentially MIS-C.

## Materials and Methods

### xenoAMPs Screening.

A previously trained SVM-based AMP classifier is used to search for AMP-like sequences (xenoAMPs) in SARS-CoV-2 proteins (GenBank accession ID: MN938384). Each protein is first scanned with a moving window with the size of 24-34 amino acid long. The classifier analyzes each query sequence and outputs a σ score that correlates with the probability of that sequence being an AMP: A σ score > 1.1 suggests a high probability (P(+1) > 0.98), while a negative σ score suggests a low probability (P(+1) < 0.50). The details of the SVM, including the training dataset, physiochemical descriptors, and training process, have been published previously (28). The results from one nonstructural protein (ORF1ab polyprotein) and two structural proteins (spike protein and membrane protein) from SARS-CoV-2 are presented in details. Representative high-scoring xenoAMP sequence from the three SARS-CoV-2 proteins are as follows:

xenoAMP(ORF1ab) from the ORF1ab polyprotein (1972 to 2004):
YKHYTPSFKKGAKLLHKPIVWHVNNATNKATYK
(σ score = 2.46, *P* (+1) = 1.00).
xenoAMP(S) from the spike protein (529 to 558):
KSTNLVKNKCVNFNFNGLTGTGVLTESNKK
(σ score = 1.52, *P* (+1) = 0.99).
xenoAMP(M)I from the membrane protein (146 to 169):
RGHLRIAGHHLGRCDIKDLPKEIT
  (σ score = 1.19, *P* (+1) = 0.98).

The 3D structures of SARS-CoV-2 ORF1ab polyprotein nsp3 (ID: 6YWL) and spike protein (ID: 6VYB) are downloaded from the RCSB PDB database. The 3D structure of SARS-CoV-2 membrane protein is predicted with RaptorX (http://raptorx.uchicago.edu/ContactMap/) (64), a webserver uses an ultradeep convolutional residual neural network to predict the local structure of a query protein sequence. ChimeraX is used to visualize and edit the protein ribbon diagram.

### Antimicrobial RDA.

The antimicrobial activity of SARS-CoV-2-derived xenoAMPs is evaluated at pH 7.5 and 5.5 with the well-established ultrasensitive RDA (30, 65). Two representative bacterial species are studied: LAC-USA300 is a well-characterized MRSA strain (66). PA01 is a *P. aeruginosa* strain with extensive drug resistance (67). Colonies are picked from the freshly streaked LB broth agar and then transferred to the liquid LB broth, which is cultured at 37 °C overnight on a shaker until the bacteria grow to stationary phase. Cultures are diluted (1:100) into fresh LB broth and incubated at 37 °C for 2 to 3 h until the bacteria reach the mid-log phase (OD600 = 0.4 to 0.6). Next, 10^6^ CFU/mL bacteria is evenly inoculated onto warm, liquid-buffered molecular-grade agarose plates adjusted to pH 7.5 (PIPES buffer) or pH 5.5 (MES buffer). When solidified, 10 µg xenoAMP(S), RP-1 or γ-RP-1 is introduced to wells of the seeded matrix and incubated at 37 °C for 3 h, at which time nutrient agar overlay medium is applied. RP-1 or γ-RP-1 are two peptides with validated antimicrobial activity (68–70). The plate is incubated at 37 °C for 24 h before the diameter of the zone of inhibition is measured. The reported diameter is the average from four independent experiments.

### Sequence Alignment.

The following procedure is used to compare xenoAMP sequences from SARS-CoV-2 to homologs in other human coronaviruses. Clustal Omega is used to align SARS-CoV-2 proteins with corresponding proteins from five other human coronaviruses, including SARS-CoV (accession ID: NC_004718), HCoV-HKU1 (accession ID: DQ415908), HCoV-OC43 (accession ID: MW532115), HCoV-229E (accession ID: NC_002645), and HCoV-NL63 (accession ID: NC_005831). The alignment results are visualized and analyzed with Jalview. The phylogenetic tree is calculated from distance matrices determined by PAM250 score using the Average Distance algorithm.

### Protease Cleavage Sites Prediction.

Prediction of the protease cleavage sites close to both the N and C terminus of the studied xenoAMPs [xenoAMP(ORF1ab), xenoAMP(S), and xenoAMP(M)] is performed using the protease specificity prediction server (PROSPR, https://mybiosoftware.com/prosper-protease-substrate-specificity-webserver.html) (71). The PROSPER server utilizes a support vector regression and biprofile Bayesian feature extraction approach to predict the cleavage sites for 24 different proteases, covering the four major protease families—Aspartic, Cysteine, Metallo, and Serine protease. The results are further validated with the protease information registered in the MEROPS database.

### Mass Spectrometry Analysis to Tracheal Aspirate Samples from COVID-19 ICU Patients.

Tracheal aspirate samples are collected from mechanically ventilated COVID-19 ICU patients who develop hypoxic respiratory failure at the University of North Carolina Medical center under an approved protocol with informed consent (University of North Carolina Chapel Hill IRB 20-0822). The median age of the enrolled patients is 59 (IQR 44 to 67) with 56% male and 44% female. The tracheal aspirate sample is collected by a deep suction catheter in an endotracheal tube. SARS-CoV-2 infection was confirmed by RT-PCR. Samples are processed in a BSL2^+^ facility. Crude tracheal aspirate has undergone a virus inactivation procedure at a final concentration of 8 M urea prior to storage at −80 °C. Samples are stored for 3 to 9 mo before being processed and analyzed at Pacific Northwest National Laboratory (PNNL). The shipment is prepared with dry ice. At PNNL, proteins are extracted along with lipids and metabolites using a modified Folch extraction following a published protocol (72). The extraction buffer contains a mixture of chloroform, methanol, water at a ratio of 8:4:3. The protein fraction that is sandwiched between the organic and aqueous phase is isolated and digested with trypsin, and peptides are analyzed using a 120-min gradient on reverse phase liquid chromatography–tandem mass spectrometry using a Waters nanoEquity™ UPLC system (Millford) coupled with a Q-Exactive mass spectrometer (Thermo Scientific) (73). The selection of peptide ion is controlled by a data-dependent acquisition mode, and the signal is analyzed by a label-free relative quantification approach. Mass spectrometric raw data are analyzed using MaxQuant label-free quantification by searching against the Homo sapiens and SARS-CoV-2 UniProt proteomes (downloaded in October 2020).

### Peptide Synthesis.

Peptides studied in this project were purchased from LifeTein with high purity (96%). All synthesized peptides have C-terminal amidation. Moreover, residual trifluoroacetic acid is substituted with hydrochloric acid before lyopholization. The following sequences have been synthesized:

xenoAMP(ORF1ab): YKHYTPSFKKGAKLLHKPIVWHVNNATNKATYK-NH_2_ (Mw: 3882.9 g/mol); xenoAMP(S): KSTNLVKNKCVNFNFNGLTGTGVLTESNKK-NH_2_ (Mw: 3254.75 g/mol); xenoAMP(M): RGHLRIAGHHLGRCDIKDLPKEIT-NH_2_ (Mw: 2734.75 g/mol); Control(ORF1ab): DDLYVKRYERGCITFGKPVIWLSHEKASLNSLT-NH_2_ (Mw: 3840.4 g/mol); Control(S): ANTEIELGVCVNYDLYGISGQGIFVEVNAT-NH_2_ (Mw: 3189.5 g/mol); Spike protein (948 to 964): LQDVVNQNAQALNTLVK-NH2 (Mw: 1867.1 g/mol).

### High-Molecular-Weight Polyinosine: Polycytidylic Acid (Poly(I:C)).

Poly(I:C) is purchased from InvivoGen. Poly(I:C) is often used as a synthetic analog of dsRNA to activate TLR3 and cytosolic RNA sensor MDA5. Although Poly(I:C) only contains two types of nucleotides (inosine and cytidine), while dsRNA contains four (adenine, cytosine, guanine, and uracil). The recognition of Poly(I:C) and dsRNA by TLR3 can be quite similar as this is not known to be a sequence-dependent process. TLR3 activation has been known to exhibit a dependence on the length of dsRNA. In our experiments, we use the same length of poly(I:C) throughout. Moreover, since the average length of SARS-CoV-2 generated dsRNA is unknown (and since that is not the only source of dsRNA in a viral infection), we have chosen to use poly(I:C) with a molecular weight in the range of 1.5 to 8 kb, which is close to the replicative form of dsRNA in picornavirus, a (+) ssRNA virus in the same class of SARS-CoV-2.

### Small-Angle X-Ray Scattering (SAXS).

SAXS is performed to analyze the crystal structure of peptide-poly(I:C) complexes. Samples are prepared following the previously published protocols (21). Poly(I:C) solution is prepared at 10 mg/g by dissolving lyophilized powder in 145 mM NaCl solution. The solution is heated up to 65 to 70 °C for 10 min before incubating at room temperature for 1 h to ensure complete annealing. XenoAMPs solutions, including those for xenoAMP(ORF1ab), xenoAMP(S), and xenoAMP(M), are made by dissolving lyophilized powder in ultrapure (18 Mohm) water at 10 mg/g. The Control(ORF1ab) and Control(S) solutions are made by dissolving lyophilized powder in dimethyl sulfoxide (DMSO) at 10 mg/g. XenoAMPs-poly(I:C) complexes are formed by mixing xenoAMPs and poly(I:C) at the isoelectric ratio in pH 7.4 10 mM HEPES buffer with 140 mM NaCl. The Control(ORF1ab) peptide and the Control(S) peptide are mixed with poly(I:C) using molar ratios equivalent to those used for the corresponding xenoAMPs_._ The net charge of all peptides is calculated using Prot-pi (https://www.protpi.ch/Calculator/ProteinTool). Samples are sealed in quartz capillaries (diameter = 1.5 mm, Hilgenberg GmbH). SAXS experiments are conducted at Stanford Synchrotron Radiation Lightsource (SSRL, Beamline 4-2) (74) using monochromatic X-rays of wavelength λ = 1.378 Å (energy 9 keV). A Pilatus3 X 1 M detector (pixel size 172 μm) is used to collect the scattering signal. Samples are spun down to form packed pellets before imaging. Four independent measurements are performed on each sample to ensure data quality. The two-dimensional powder diffraction pattern is integrated with the Nika package 1.81 in Igor Pro 7. The integrated intensity I(*q*) is plotted against the *q*, where *q* is the magnitude of the scattering vector defined as $q=\frac{4\pi \;sin\theta }{\lambda }$ with θ the scattering angle and λ the wavelength of the X-ray. In cases when only one strong diffraction peak is observed, the average interplanar distance is calculated as $d=\;\frac{2\pi }{q}$ . The domain size of the nanocrystalline structure is calculated following previous work (21). The measured diffraction signal is first background subtracted before fitting with a Lorentzian line shape. The equation used is:

$$\begin{array}{c} S\left(q\right)=\frac{w^{3}}{4\pi {\left(lq-q_{0}l^{2}\;+\;{\left(\frac{w}{2}\right)}^{2}\right)}^{2}}, \end{array}$$

where *q*_0_ is the position of the peak, and *w* is the peak width. The extracted value of *w* is correlated with the linear domain size *L* in a squared-Lorentzian line shape using warren approximation.:

$$L=\frac{{\left(8\pi \right)}^{1/2}}{\frac{w}{2}}$$

### Human Cell Culture and Activation.

HDMVEC and HAoEC are purchased from PromoCell. NHEKs are purchased from Life Technologies. THP-1 human monocyte, BEAS-2B human bronchial epithelial cells, and human umbilical vein endothelial cell–derived immortalized EA.hy926 cells are purchased from ATCC. All the cells are maintained at 37 °C in a 5% CO_2_ incubator. The number of biologic replicates in each experiment is noted in the figure legend.

HDMVECs and HAoECs are maintained in Endothelial Cell Growth Medium MV (PromoCell). Cells are seeded in a 24-well plate at a density of 2.5 × 10^5^ cells/well a day before the treatment. HDMVECs are stimulated for 24 h with 2.5 µg/mL poly(I:C) alone or complexed with xenoAMPs at 2× isoelectric ratio, while HAoECs are treated by 1.25 µg/mL poly(I:C) and the corresponding controls. The released IL-6 in the supernatant is quantified with ELISA (R&D Systems).

NHEK cells are maintained in EpiLife medium (ThermoFisher) with added human keratinocyte growth supplement (ThermoFisher). Cells are seeded in 96-well tissue culture–treated plates at a density of 0.25 × 10^5^ cells/well overnight before the treatment. NHEK cells are stimulated for 24 h with 2.5 µg/mL poly(I:C) alone or complexed with xenoAMPs at 2× isoelectric ratio, while HAoECs are treated by 2.5 µg/mL poly(I:C) and the corresponding controls. The released IL-6 in the supernatant is quantified with ELISA (R&D Systems).

THP-1 cells are maintained in complete Roswell Park Memorial Institute (RPMI)-1640 media supplement with 10% fetal bovine serum (FBS), 100 U/mL penicillin–streptomycin, and 0.05 mM beta-mercaptoethanol. Prior to the experiment, cells are cultured in serum-free RPMI-1640 medium for 12 h before seeding into a 96-well plate. Cells (1 × 10^6^ cells/well) are stimulated for 18 h with 5 µg/mL poly I:C (InvivoGen) alone or complexed with xenoAMPs at 2× isoelectric ratio. The supernatant is collected by spinning down cells at 3,000 rpm for 1min. The released IL-8 is quantified by ELISA. (Antibodies are purchased from R&D Systems).

BEAS-2B cells are cultured in Airway Epithelial Cell Growth Medium (PromoCell). The cells are seeded in 96-well plates at a density of 2.5 × 10^4^ cells/well a day before the experiment and were stimulated for 24 h in serum‐free media with 5 µg/mL poly (I:C) (Tocris) alone or complexed with xenoAMPs at isoelectric ratio. The secreted IL-6 is quantified with ELISA. (DuoSet, R&D Systems).

EA.hy926 cells were cultured with Dulbecco’s Modified Eagle’s Medium (DMEM) supplement with 10% FBS and 1% PSF. Cells were seeded into a 24‐well plate (2.5 × 10^5^ cells/well) with DMEM supplement with 1% FBS and 0.1% PSF a day before stimulation. The next day, cells were stimulated for 24 h in serum‐free media with 5 µg/mL poly (I:C) (Tocris) alone or complexed with xenoAMPs at the isoelectric ratio. IL-6 and IL-8 levels in the supernatant were measured by ELISA (Duo‐Set, R&D Systems, MN). To quantify TF activity, after 6 h of stimulation, cells are lysed with 15 mM octyl-β-d-glucopyranoside in 25 mM HEPES/saline buffer at 37 °C for 15 min, and the lysates were tested for TF activity in a two-stage Factor Xa activity assay as previously described (75). Innovin was used to generate a standard curve.

### RT-qPCR Analyses.

RT-qPCR is used to determine the mRNA abundance in HDMVEC. Total cellular RNA is extracted using the PureLink RNA Mini Kit (Life Technologies Corporation). One hundred nanograms of mRNA is reverse transcribed to cDNA using the Verso cDNA Synthesis Kit (Thermo Fisher Scientific Inc). Quantitative, real-time PCR is performed on the CFX96 real-time system (Bio-Rad) using predeveloped TaqMan gene expression assay (Applied Biosystems) or SYBR Green Mix (Bimake). The housekeeping gene TBP (TATA-binding box protein) is used to normalize gene expression in samples. Specific primer sequences are shown in *SI Appendix*, Table S3.

### RNA Sequencing and Data Analysis.

Purified RNA from the HDMVEC is submitted to the University of California, San Diego (UCSD) Institute for Genomic Medicine core facility for library preparation and high-throughput next-generation sequencing. Libraries are constructed using TruSeq Stranded mRNA Library PrepKits (Illumina, San Diego, CA) and run on a HiSeq. 2500 instrument (Illumina). Sequence reads are quantitated using Whippet (version 1.6.1) (76) with the GRCh38.p13 RefSeq genome annotations. Differential expression analysis and PCA analysis are performed with DESeq2 (version 1.34.0) (77). KEGG pathway analysis is performed with significant differentially expressed genes (adjusted *P*-value < 0.05) using clusterProfiler (version 4.2.2) (78–80).

### Mouse BMDM Culture and Activation.

Mouse bone marrow cells are collected from C57Bl/6J mice (at 6~8 wks) following the animal protocol approved by UCLA institutional animal care and use committee (IACUC). Bone marrow cells are extracted from the femur and tibia and maintained in RPMI supplement with 100 U/mL penicillin–streptomycin (Gibco) and 20 mM L-glutamine (Gibco) for 7 d. To differentiate the cells into macrophages, 50 µg/mL MCSF (Gibco) is added. On the day before the experiment, the BMDMs are seeded into a 24-well plate at a density of 5 × 10^5^ cells/well. Cells are stimulated for 24 h with 2.5 µg/mL poly(I:C) (InvivoGen) alone or poly(I:C) complexed with xenoAMPs at the isoelectric ratio. The released IL-6 is quantified with ELISA (Invitrogen).

### In Vivo Immune Activation.

Eight‐ to ten‐week‐old male C57Bl/6J mice are administered with poly(I:C) (100 µg/mouse) or poly(I:C)-xenoAMP(ORF1ab) complex [100 µg poly(I:C) and 344 µg xenoAMP(ORF1ab) per mouse] in a final volume of 200 µL by retro-orbital injection. Five hundred microliters of blood is collected via inferior vena cava puncture after injecting 200 µL of the sodium citrate 2h post treatment. Total WBC, neutrophil, and lymphocyte and monocyte counts are determined with an Element HT5 veterinary hematology analyzer (Heska, Loveland, CO). Plasma is prepared by centrifugation of citrated whole blood at 4,500 × g for 15 min at room temperature (75). Levels of mouse CXCL-1 and IL-6 in plasma are measured by ELISA (Duo‐Set, R&D Systems, MN). All experimental animal procedures are approved by the IACUC at University of North Carolina at Chapel Hill.

### Statistical Analysis.

The data were plotted and analyzed with GraphPad 8.

All of the measurements are performed on distinct samples. Significance is calculated with one-way ANOVA with Tukey correction or two-sided unpaired *t* test. A 95% confidence interval is used. *P* < 0.05 is considered a significant difference. All the data are represented as mean ± SD. The panels in each figure are arranged in Adobe Illustrator.

## Supplementary Material

Appendix 01 (PDF)

## Data Availability

The proteomics data from the tracheal aspirate samples from COVID-19 ICU patients have been deposited at massive.ucsd.edu (Dataset Identifier: MSV000089614). RNA sequencing data from HDMVEC have been deposited at Gene Expression Omnibus (accession ID: GSE205487). The computer code used in the machine learning screening of AMP mimicking sequence has been reported in our previous publication (24). The code for analyzing and plotting the RNA sequencing data has been deposited at GitHub (https://github.com/Yuezhangcv/xenoAMP-data-code.git).

## Footnotes

^*^In addition to the high charge, LL-37 is characterized by well-defined facial amphiphilicity, which allows assembly into superhelical protofibrils with sufficient linear charge density to organize nucleic acids.
