# Supplementary material for: Viral afterlife: SARS-CoV-2 as a reservoir of immunomimetic peptides that reassemble into proinflammatory supramolecular complexes
Source: Proc Natl Acad Sci U S A. 2024 Feb 2;121(6):e2300644120. doi: 10.1073/pnas.2300644120 (PMC10861912; doi:10.1073/pnas.2300644120)
Supplement: Supplementary file 1 — Appendix 01 (PDF) [file pnas.2300644120.sapp.pdf]

## Supplemental figures

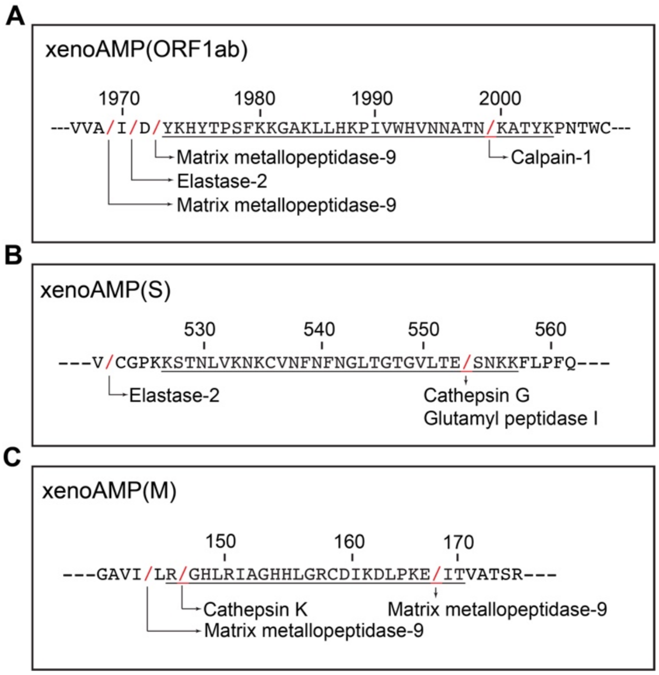


**Fig. S1. | *In silico* prediction of the protease cleavage sites close to the N and C termini of the SARS-CoV-2 xenoAMPs.** The underlined sequences are the selected xenoAMPs. The red forward slash indicates the protease cleavage site.

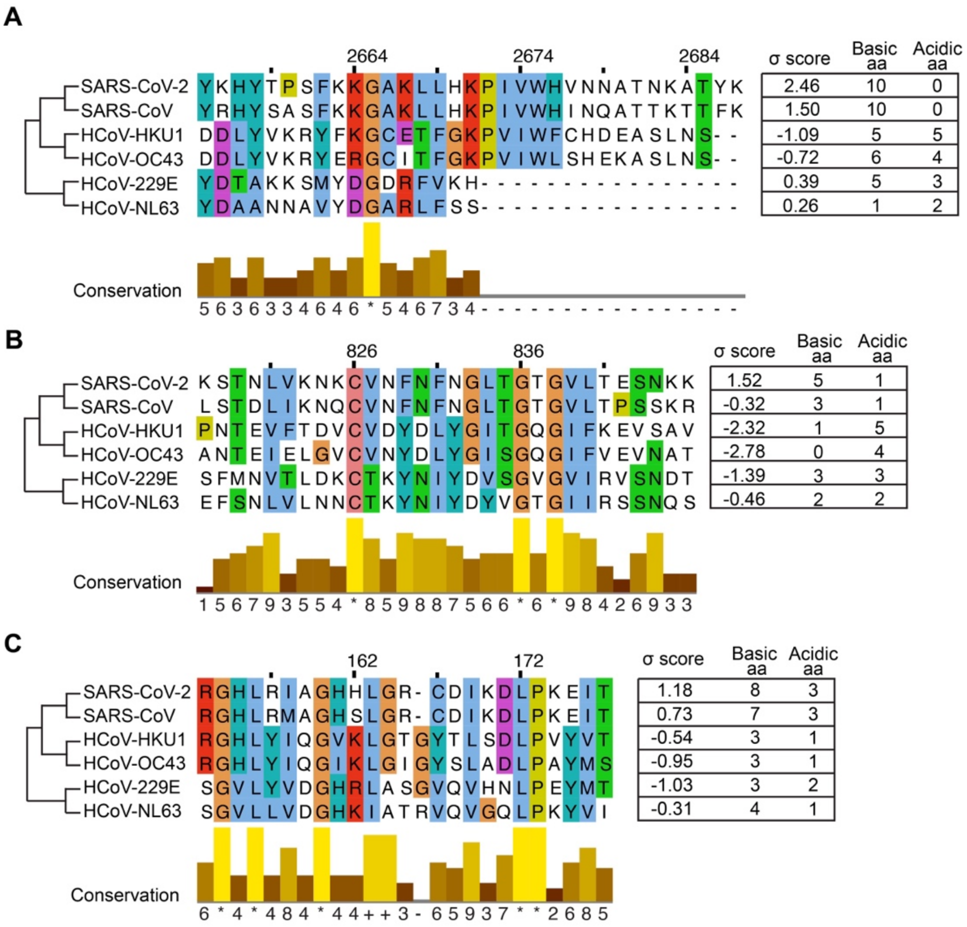


**Fig. S2. | Sequence alignment of six human coronaviruses.** The SARS-CoV-2 ORF1ab protein, spike protein, and membrane protein are aligned with the corresponding proteins from five human coronaviruses, with one highly pathogenic one (SARS-CoV) and four less pathogenic ones (HCoV-HKU1, HCoV-OC43, HCoV-229E and HCoV-NL63). The phylogenetic tree shows the evolutionary relationship between these 6 human coronaviruses. The whole alignments are zoomed in to where xenoAMP(ORF1ab), xenoAMP(S) and xenoAMP(M) are derived. Color is assigned to each residue using the ClustalX color scheme. Each amino acid position is scored between 0-11 to show the degree of conservation. 11(+) means fully conserved. 10(*) means the properties are conserved. 0(-) means not conserved. σ denotes the AMPness. A sequence with large, positive σ is AMP-like sequence, while a sequence with large, negative σ score does not mimic AMP. The number of basic amino acids (Lysine(K), arginine(R) and histidine(H)) and acidic aa (aspartic acid(D) and glutamic acid(E)) are shown in the chart.

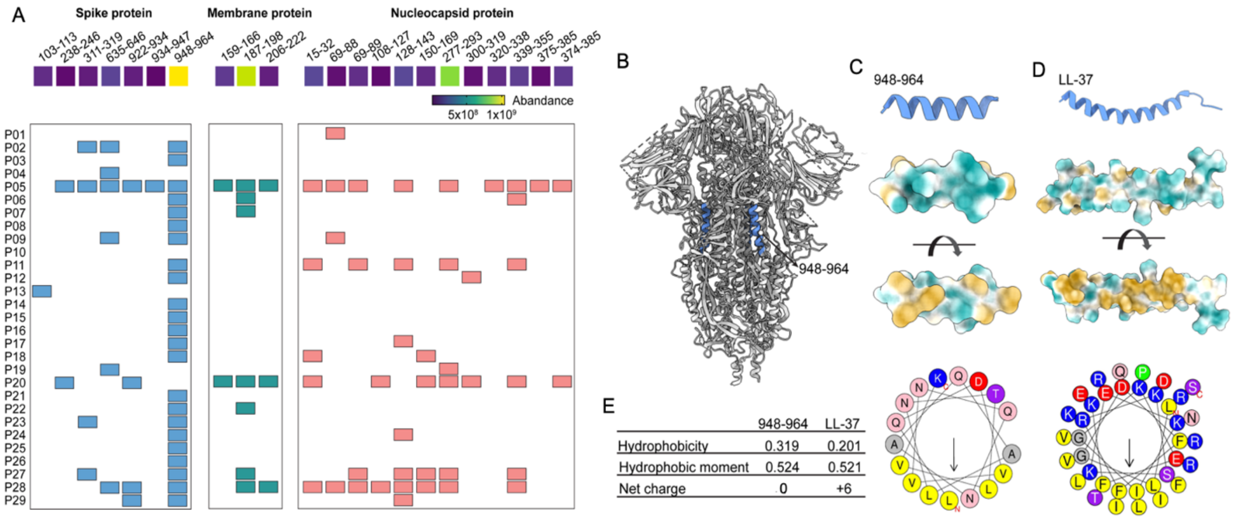


**Fig. S3 | Mass spectrometry measurement of tracheal aspirate samples from critical COVID-19 patients.** To determine whether any SARS-CoV-2 proteins and protein fragments are found in host biofluids and thereby exposed to diverse cell types, and to assess whether such fragments are potentially harmful from their overlap with xenoAMPs, we apply liquid chromatography-tandem mass spectrometry (LC–MS/MS to analyze protein fragments in tracheal aspirate samples from 29 critically ill COVID-19 patients with hypoxic respiratory failure. (P1-P29) We have chosen to use trypsin as the protease used to enable the mass spectrometry measurements, The reason is that trypsin cleavage sites are the same as those from trypsin-like serine proteases already present in the host innate immune system. The general expectation is that some of the highest scoring AMP-like fragments will bind strongly to nucleic acids and lipids, since such binding is related to their antimicrobial mechanism of action. Consequently, the highest scoring sequences may not be observable in isolation. Furthermore, we do not want to bias our analysis of the proteome by pre-selecting the peptides to be detected in tracheal aspirate from ICU patients. Therefore, we have performed bottom-up proteomics analysis rather than bias the proteomics toward the detection of specific peptides "predicted" to be AMP-like by machine learning. Finally, our analysis is performed in data dependent acquisition (DDA) mode: during the chromatographic elution only the most abundant peptides were fragmented to obtain sequence information, which implies that only the most abundant peptides that remain in isolation are detected. We investigate whether any xenoAMPs or fragments of xenoAMPs can be observed under these conditions, and in how many patients. As a comparison, we perform a similar study for LL-37, a host AMP expected to be produced during neutrophilia, one of the inflammatory consequences in COVID-19.

Consistent with previous observations from gargle lavage fluid and nasopharyngeal swabs, SARS-CoV-2 peptide fragments are observed: 7 peptides from the spike protein (sequence coverage (SC) of 7.38%), 3 peptides from the membrane protein (SC of 16.6%), 12 peptides from the nucleocapsid protein (SC of 42.95%). **(A)** One of the most abundant fragments, 948-964 (LQDVVNQNAQALNTLVK), is a peptide fragment originated from spike protein heptad repeat 1(HR1), which adopts an alpha-helical conformation with a large hydrophobic face on one side and hydrophilic face on the other side. **(B-E)** Using SAXS, we show that this fragment can remodel membranes the same way that host AMPs do, via generation of negative Gaussian curvature topologically necessary for pore formation. The fragment induces a Pn3m cubic phase with a lattice constant of 17.7nm with a peptide/lipid molar ratio of 1/72 (data not shown). As a calibrating readout, fragments of LL-37, a host AMP generated during the inflammatory process, are found in 20 of the 29 patient samples. In comparison, SARS-CoV-2 peptide fragments are found in 28 of the 29 patient samples, with some of the fragments having high enough scores to be xenoAMPs themselves.

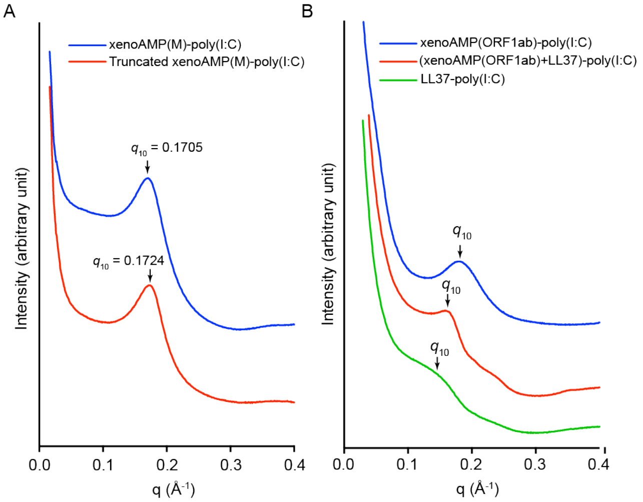


C

| Samples | Peak Width(w, Å) | Domain size (L,nm) |
|---|---|---|
| xenoAMP(ORF1ab)-poly(I:C) | 0.07804 | 6.4 |
| (xenoAMP(ORF1ab)+LL37)-poly(I:C) | 0.03187 | 15.7 |
| LL37-poly(I:C) | 0.06616 | 7.6 |

**Fig. S4 | Assembly of pro-inflammatory SARS-CoV-2 xenoAMP-poly(I:C) complexes under non-ideal conditions: (A)** Variation of peptide length from non-ideal cleavage: Complexes from incubating xenoAMP(M) (RGHLRIAGHHLGRCDIKDLPKEIT, σ=1.19) with poly(I:C) and complexes from the truncated version of xenoAMP(M) (GHLRIAGHHLGRCDIKDLPK, σ=1.32) with poly(I:C), both at the isoelectric peptide to nucleic acid stoichiometry, are measured with SAXS. Strong pro-inflammatory structural signatures are seen in both cases regardless of truncation, with the former having a dsRNA spacing at 3.68nm, and the latter at 3.64nm. **(B, C)** A comparison of peptide-dsRNA complexes formed using xenoAMP(ORF1ab) only, form using host LL-37 only, and formed using a 1:3 mixture of xenoAMP(ORF1ab) and LL-37. While all complexes exhibit the pro-inflammatory poly(I:C) spacing around 3.3-3.7nm, the mixed xenoAMP-LL-37 complex is the most ordered with additional higher order peaks, with a sharper linewidth and larger domain size.

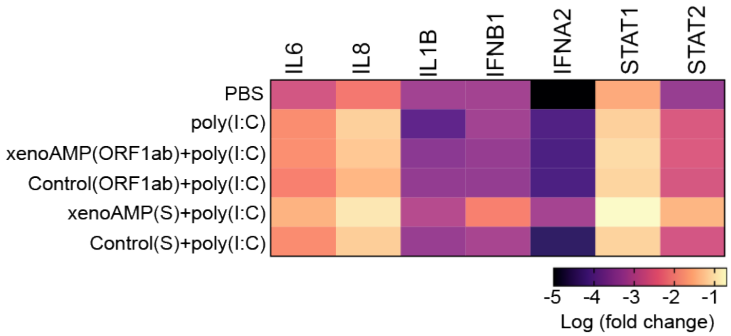


**Fig. S5. |** Gene expression change of the pro-inflammatory cytokines (IL6, IL8 and IL1B) and Interferon (IFN) pathway-related genes (IFNB1, IFNA2, STAT1 and STAT2) in HDMVEC cells measured by Quantitative reverse transcription PCR (RT-qPCR). The fold of change is calculated by normalizing the counts against the TATA box binding protein (TBP). (n=4)

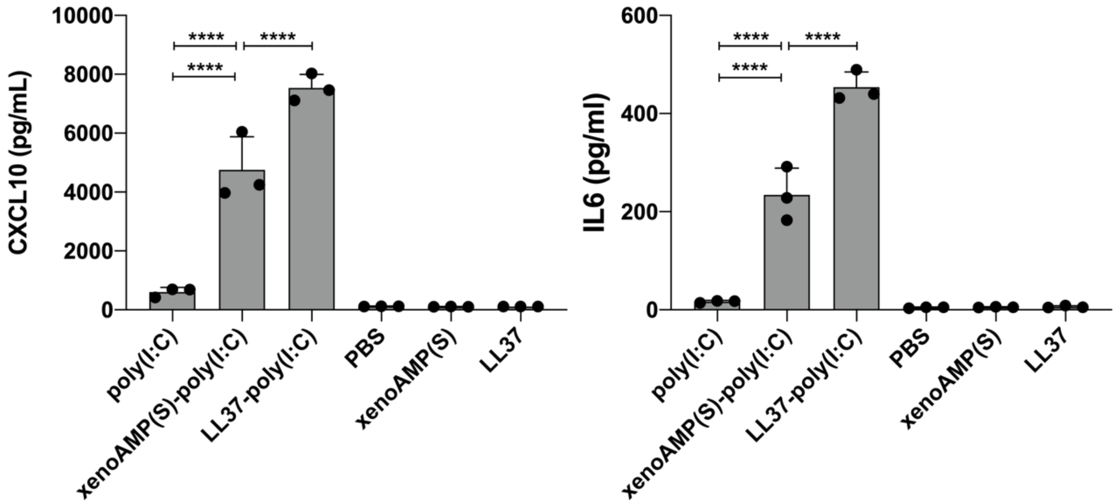


**Fig. S6. xenoAMPs-**poly(I:C) **and LL37-**poly(I:C) **complexes both activate cytokine production in primary Normal Human Epidermal Keratinocytes (NHEK).** NHEK cells at passage 3 are incubated with 2.5 µg/mL poly(I:C), xenoAMP- poly(I:C) complexes, LL37- poly(I:C) complexes, and the related controls for 24 hours. The CXCL10 and IL-6 concentration is quantified with ELISA (n=3). Data are presented as mean ± SD. The statistical analysis is performed using one-way ANOVA. (*p<0.05, **p<0.01, ***p<0.001, ****p<0.0001)

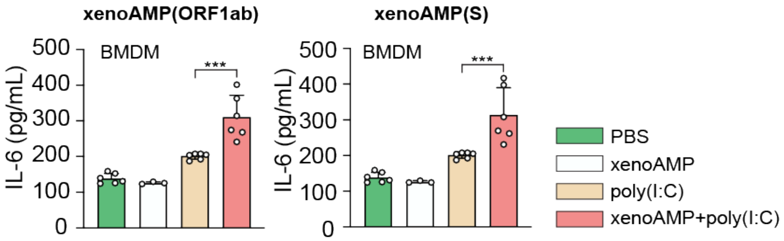


**Fig. S7 |** Mouse bone marrow derived macrophages (BMDM) are treated by xenoAMP-poly(I:C), poly(I:C), or associated controls. The released IL-6 in the supernatant is measured with ELISA (n=6). Data are presented as mean ± SD. The statistical analysis is performed with one-way ANOVA. (***$p$<0.001)

## Supplement tables

**Table. S1 | Antimicrobial test of SARS-CoV-2 xenoAMP(S) against *S. aureus* and *P. aeruginosa*.**

| Pathogen (Strain) | Peptide | PIPES (pH 7.5) | | | | MES (pH 5.5) | | | | Mean (StdDev) | | | |
|---|---|---|---|---|---|---|---|---|---|---|---|---|---|
| | | | | | | | | | | pH 7.5 | SD | pH 5.5 | SD |
| *S. aureus* (LAC-USA300) | RP-1 | 15.02 | 14.27 | 14.94 | 15.20 | 9.38 | 9.08 | 10.22 | 9.35 | 14.86 | 0.41 | 9.51 | 0.49 |
| | γ-RP-1 | 9.44 | 10.96 | 9.36 | 12.35 | 4.88 | 4.83 | 3.12 | 5.86 | 10.53 | 1.42 | 4.67 | 1.14 |
| | xenoAMP(S) | 7.93 | 5.00 | 5.04 | 6.40 | 0.00 | 0.00 | 0.00 | 0.00 | 6.09 | 1.39 | 0.00 | 0.00 |

| Pathogen (Strain) | Peptide | PIPES (pH 7.5) | | | | MES (pH 5.5) | | | | Mean (StdDev) | | | |
|---|---|---|---|---|---|---|---|---|---|---|---|---|---|
| | | | | | | | | | | pH 7.5 | SD | pH 5.5 | SD |
| *P. aeruginosa* (PA01) | RP-1 | 13.59 | 13.94 | 14.33 | 13.80 | 11.94 | 13.45 | 11.02 | 12.01 | 13.92 | 0.31 | 12.11 | 1.00 |
| | γ-RP-1 | 9.14 | 11.27 | 10.84 | 11.05 | 6.99 | 7.98 | 8.39 | 7.96 | 10.58 | 0.97 | 7.83 | 0.59 |
| | xenoAMP(S) | 8.25 | 8.03 | 6.85 | 6.44 | 7.10 | 8.08 | 3.90 | 3.82 | 7.39 | 0.88 | 5.73 | 2.19 |

**Table S2. | Persistence of AMP-like characteristics fir SARS-CoV-2 fragments of different lengths close to the three exemplar xenoAMPs we study:** It can be seen in all 3 cases that the AMP characteristics remain quite persistent for various imperfect cleavage events that do not produce the exact xenoAMPs we study in detail. Structural parameters, such as the detailed distribution of hydrophobicity and high cationic charge, are important, but these are partially encoded into the sigma score since they are typical AMP characteristics.

| Length | Sequences | σ score | P(+1) | Note |
|---|---|---|---|---|
| 35 | YKHYTPSFKKGAKLLHKPIVWHVNNATNKATYKPN | 2.57 | 1.00 | |
| 34 | YKHYTPSFKKGAKLLHKPIVWHVNNATNKATYKP | 2.57 | 1.00 | |
| 33 | YKHYTPSFKKGAKLLHKPIVWHVNNATNKATYK | 2.46 | 1.00 | xenoAMP(ORF1ab) |
| 32 | YKHYTPSFKKGAKLLHKPIVWHVNNATNKATY | 2.23 | 1.00 | |
| 31 | YKHYTPSFKKGAKLLHKPIVWHVNNATNKAT | 2.24 | 1.00 | |
| 30 | YKHYTPSFKKGAKLLHKPIVWHVNNATNKA | 2.20 | 1.00 | |
| 29 | YKHYTPSFKKGAKLLHKPIVWHVNNATNK | 2.12 | 1.00 | |
| 28 | YKHYTPSFKKGAKLLHKPIVWHVNNATN | 1.02 | 0.97 | |
| 27 | YKHYTPSFKKGAKLLHKPIVWHVNNAT | 0.93 | 0.96 | |
| 26 | YKHYTPSFKKGAKLLHKPIVWHVNNA | 0.97 | 0.96 | |
| 25 | YKHYTPSFKKGAKLLHKPIVWHVNN | 0.93 | 0.97 | |
| 24 | YKHYTPSFKKGAKLLHKPIVWHVN | 1.02 | 0.99 | |

| Length | Sequences | σ score | P(+1) | Note |
|---|---|---|---|---|
| 32 | KSTNLVKNKCVNFNFNGLTGTGVLTESNKKFL | 1.55 | 0.99 | |
| 31 | KSTNLVKNKCVNFNFNGLTGTGVLTESNKKF | 1.53 | 0.99 | |
| 30 | KSTNLVKNKCVNFNFNGLTGTGVLTESNKK | 1.52 | 0.99 | xenoAMP(S) |
| 29 | KSTNLVKNKCVNFNFNGLTGTGVLTESNK | 1.33 | 0.99 | |
| 28 | KSTNLVKNKCVNFNFNGLTGTGVLTESN | 0.18 | 0.65 | |
| 27 | KSTNLVKNKCVNFNFNGLTGTGVLTES | 0.34 | 0.76 | |
| 26 | KSTNLVKNKCVNFNFNGLTGTGVLTE | 0.39 | 0.79 | |
| 25 | KSTNLVKNKCVNFNFNGLTGTGVLT | 0.62 | 0.89 | |
| 24 | KSTNLVKNKCVNFNFNGLTGTGVL | 0.66 | 0.90 | |
| 23 | KSTNLVKNKCVNFNFNGLTGTGV | 1.07 | 0.97 | |
| 22 | KSTNLVKNKCVNFNFNGLTGTG | 1.11 | 0.98 | |
| 21 | KSTNLVKNKCVNFNFNGLTGT | 1.20 | 0.98 | |

| Length | Sequences | σ score | P(+1) | Note |
|---|---|---|---|---|
| 26 | RGHLRIAGHHLGRCDIKDLPKEITVA | 0.81 | 0.94 | |
| 25 | RGHLRIAGHHLGRCDIKDLPKEITV | 0.88 | 0.95 | |
| 24 | RGHLRIAGHHLGRCDIKDLPKEIT | 1.19 | 0.98 | xenoAMP(M) |
| 23 | RGHLRIAGHHLGRCDIKDLPKEI | 1.22 | 0.98 | |
| 22 | RGHLRIAGHHLGRCDIKDLPKE | 1.03 | 0.97 | |
| 21 | RGHLRIAGHHLGRCDIKDLPK | 1.35 | 0.99 | |
| 20 | RGHLRIAGHHLGRCDIKDLP | 1.01 | 0.97 | |
| 19 | RGHLRIAGHHLGRCDIKDL | 1.00 | 0.96 | |
| 18 | RGHLRIAGHHLGRCDIKD | 1.13 | 0.98 | |
| 17 | RGHLRIAGHHLGRCDIK | 1.38 | 0.99 | |
| 16 | RGHLRIAGHHLGRCDI | 1.01 | 0.97 | |
| 15 | RGHLRIAGHHLGRCD | 0.74 | 0.92 | |

*P*(+1) denotes the probability of a sequence with the physiochemical features resembling natural AMP.

**Table. S3 | Primers used in qPCR experiment.**

| Gene | | Sequence (5'-3') |
|---|---|---|
| TBP | Forward | TGTATCCACAGTGAATCTTGGTTG |
| | Reverse | GGTTCGTGGCTCTCTTATCCTC |
| IL6 | Forward | ACTCACCTCTTCAGAACGAATTG |
| | Reverse | CCATCTTTGGAAGGTTCAGGTTG |
| IL8 | Forward | GAGAGTGATTGAGAGTGGACCAC |
| | Reverse | CACAACCCTCTGCACCCAGTTT |
| IL1b | Forward | AGCTACGAATCTCCGACCAC |
| | Reverse | CGTTATCCCATGTGTCGAAGAA |
| IFNb1 | | Hs01077958_s1 |
| IFNb2 | | Hs00265051_s1 |
| Stat1 | | Hs01013996_m1 |
| Stat2 | | Hs01013115_g1 |
